# Academic Procrastination and Negative Emotions Among Adolescents During the COVID-19 Pandemic: The Mediating and Buffering Effects of Online-Shopping Addiction

**DOI:** 10.3389/fpsyg.2021.789505

**Published:** 2022-02-03

**Authors:** Qiaoling Wang, Ziyu Kou, Yunfeng Du, Ke Wang, Yanhua Xu

**Affiliations:** ^1^College of Resource Environment and Tourism, Capital Normal University, Beijing, China; ^2^Beijing Academy of Educational Sciences, Beijing, China; ^3^College of Teacher Education, Capital Normal University, Beijing, China; ^4^Zhanjiang No.2 Middle School, Zhanjiang, China

**Keywords:** academic procrastination, negative emotions, online-shopping addiction, adolescents, COVID-19

## Abstract

**Purpose:**

The COVID-19 pandemic that began in 2019 has had a significant impact on people’s learning and their lives, including a significant increase in the incidence of academic procrastination and negative emotions. The topic of how negative emotions influences academic procrastination has been long debated, and previous research has revealed a significant relationship between the two. The purpose of this study was to further investigate the mediating and buffering effects of online-shopping addiction on academic procrastination and negative emotions.

**Methods:**

The researchers conducted a correlation analysis followed by a mediation analysis and developed a mediation model. The study used stratified sampling and an online questionnaire as the data collection method. In this study, first, five freshmen students at vocational and technical colleges in Guangdong Province, China, were called to distribute the questionnaire. Second, after communicating with them individually, first-year students of Guangdong origin were selected as participants. Finally, 423 freshman students participated by completing the questionnaire. The questionnaire consisted of 4 parts: demographic information, an online-shopping-addiction scale, an academic-procrastination scale and a negative-emotions scale. A total of 423 students, 118 males (27.9%) and 305 females (72.1%) from 10 vocational and technical colleges in Guangdong were surveyed. SPSS 25.0 was used to process and analyze the data. The data collected were self-reported.

**Results:**

The results showed that: first, academic procrastination was significantly and positively associated with online-shopping addiction (*r* = 0.176, *p* < 0.01). Second, academic procrastination was significantly and positively associated with negative emotions (*r* = 0.250, *p* < 0.01). Third, online-shopping addiction was significantly and positively associated with negative emotions (*r* = 0.358, *p* < 0.01). In addition, academic procrastination had a significant positive predictive effect on online-shopping addiction (β = 0.1955, *t* = 3.6622, *p* < 0.001). Online-shopping addiction had a significant positive predictive effect on negative emotions (β = 0.4324, *t* = 7.1437, *p* < 0.001).

**Conclusion:**

This study explored the relationship between students’ academic procrastination, negative emotions, and online-shopping addiction during the COVID-19 pandemic. The results indicated that students’ level of academic procrastination positively influenced their level of online-shopping addiction and negative emotions, and their level of online-shopping addiction increased their negative emotions. In addition, there was a mediating effect between the degree of participants’ online-shopping addiction and their degree of academic procrastination and negative emotions during the pandemic. In other words, with the mediating effect of online-shopping addiction, the higher the level of a participant’s academic procrastination, the more likely that the participant would have a high score for negative emotions.

## Introduction

The COVID-19 epidemic has posed a serious threat to people’s health and to their lives (Zhang and Ma, 2020). As a result of the pandemic, the social networks that many people rely on have been disrupted. Many others have not had the luxury of social isolation while facing the threat of losing their jobs and even the loss of loved ones. Not surprisingly, depression and anxiety have been on the rise (Hofmann, 2021). In China, the characterization of novel coronavirus pneumonia by the National Health Commission has led to increased negative emotions such as anxiety and anger, decreased well-being, and increased sensitivity to social risks in the overall mindset of society (Yang et al., 2021). Therefore, the negative effects of COVID-19 have received widespread attention from the academic community.

Epidemics can have many negative effects on people’s mental health. One survey showed that increased fear of COVID-19 was directly associated with weakened mental health, which in turn was associated with decreased quality of life (Alyami et al., 2021). Patients infected with COVID-19 have suffered from severe psychological problems, which may negatively affect their quality of life and their sleep (Hofmann, 2021). An infection can also trigger other psychological problems, such as panic attacks, anxiety, and depression (Qiu et al., 2020). Therefore, mental-health issues have been considered as an important topic during the COVID-19 pandemic.

Academic procrastination and negative emotions have attracted the interest of researchers worldwide. *Academic procrastination* is a phenomenon whereby an individual intentionally delays some learning tasks that must be completed without regard for the possible adverse consequences. The challenges posed by the COVID-19 pandemic can increase procrastination in academic activities that a student either dislikes or is passionate about (Peixoto et al., 2021). The relationship between the two phenomena deserves more in-depth study.

According to the existing literature, many learners have experienced negative emotions during the pandemic. A common feature of procrastination is the emphasis on repairing negative emotions at the expense of pursuing other important self-control goals (Solomon and Rothblum, 1984; Ferrari et al., 1995). Sirois and Pychyl argue that as a form of self-regulation failure, procrastination has a great deal to do with short-term emotion repair and emotion regulation (Sirois and Pychyl, 2013). During the COVID-19 pandemic, levels of procrastination can mediate the link between academic anxiety and self-manipulation in part (Jia et al., 2021). However, few studies have examined the relationship between academic procrastination and negative emotions during the pandemic. In addition, learners’ online-shopping addiction is often associated with their negative emotions. Researchers have found that compulsive shoppers often use shopping as a means to change their emotion for the better. Compulsive buyers satisfy their desires by shopping when faced with the frustration associated with a lack of shopping. The pleasurable feelings associated with shopping seem to temporarily overshadow the negative effects, thus perpetuating a compulsive-shopping cycle (Clark and Calleja, 2009). However, few studies have focused on the relationship between academic procrastination and online-shopping addiction or on the mediating role of online-shopping addiction between academic procrastination and negative emotions.

To deepen the understanding of negative emotions in academic settings, this study explores the relationship between academic procrastination and negative emotions, and also the mediating role of online-shopping addiction between the two. We have divided this paper into six parts. The next part of the paper contains the literature review and hypotheses, focusing on the concepts and related research on academic procrastination, negative emotions, and online-shopping addiction and hypothesizing the relationship between these variables. The third section describes the participant, instruments, and data-analysis methods for this study. This is followed by the results of the data analysis. The fifth section is a discussion of the results and of expectations for future research, and the final part is the conclusion.

In summary, the assumptions were made: (a) a positive relationship existed between students’ academic procrastination and negative emotions; (b) a positive relationship existed between academic procrastination and online-shopping addiction; (c) a positive relationship existed between online-shopping addiction and negative emotions.

## Review of the Literature

### Academic Procrastination

*Procrastination* is to voluntarily delay an intended course of action despite expecting to be worse off for the delay (Steel, 2007). At the same time, procrastination is an irrational act of delay (Steel, 2010). Procrastination is a failure of self-regulation. And people’s attempts to regulate their emotions through procrastination can have counterproductive results (Tice and Bratslavsky, 2000). Procrastination is a form of self-regulatory failure rooted in unpleasant emotional states and an inability to control one’s behavior in pursuit of short-term emotional fixes (Stead et al., 2010; Ramzi and Saed, 2019). Steel found that procrastination is a common and harmful form of self-regulatory failure and applied temporal motivation theory to explain this self-regulatory behavior (Steel, 2007). Three researchers have illustrated the mechanisms by which procrastination occurs in terms of personal self-regulation and self-control failures. This fits in with the occurrence of adolescent procrastination that this study proposes to explore. Therefore, self-regulatory mechanisms and temporal motivation theory served as the theoretical basis for constructing this study’s model.

Some researchers have rethought the definition of procrastination and proposed that procrastination can be divided into active and passive procrastination. Passive procrastination is procrastination in its traditional sense, while active procrastination refers to planned behavior. Active procrastinators are more inclined to work under pressure and make the decision to procrastinate after deliberation (Chun Chu and Choi, 2005). Active procrastinators are more highly emotionally stable (Liu et al., 2017). Active procrastinators are comfortable with change when there is an unexpected situation. So active procrastinators may be able to work more effectively than others (Chun Chu and Choi, 2005). In addition, active procrastination can indirectly influence creative thinking through creative self-efficacy, so researchers have argued that more attention could be paid to the positive effects of active procrastination (Liu et al., 2017).

*Academic procrastination* occurs when a person is clearly aware of the need to complete an academic task but does not complete it within the expected time (Wolters, 2003). This tendency and intention of an individual to postpone learning activities is believed to be a result of post-modern values in a post-industrial society (Dietz et al., 2011). In addition, academic procrastination is related to self-regulated learning (Wolters, 2003; Kandemir, 2014). Academic procrastination in college students has a significant negative impact on their subsequent academic performance (Gareau et al., 2019). Academic procrastination also have a greater negative impact on younger students, and high levels of academic procrastination is more detrimental to academic performance (Goroshit and Hen, 2019). Academic procrastination is associated with the pursuit of perfectionism. Research suggests that levels of maladaptive perfectionism may be exacerbated if students are criticized for not meeting parental expectations. Students can develop a fear of failure, which ultimately leads to procrastination (Shih, 2016). Self-directed perfectionism and socially oriented perfectionism are two dimensions of perfectionism. Self-directed perfectionism was negatively associated with academic procrastination and socially oriented perfectionism was positively associated with procrastination (Closson and Boutilier, 2017). A study suggests that social and environmental factors also influence students’ procrastination (Nordby et al., 2017).

### Negative Emotions

*Positive* emotions and *negative* emotions are the two main and relatively independent dimensions of the human emotion structure (Diener et al., 1985). *Positive* emotions broaden people’s reserves of thinking and action and help them build sustained and long-lasting personal resources, including physical, intellectual, social, and psychological resources (Fredrickson, 2001). *Negative* emotions are subjective painful emotions and also a variety of aversive emotional states. It includes anger, contempt, disgust, guilt, fear, and tension (Watson et al., 1988). These emotions can cause a person to have difficulty concentrating, paying attention to details, and understanding information (Rowe and Fitness, 2018). Expressing positive emotions undoubtedly brings many benefits. Expressing negative emotions may also have positive outcomes: a person who expresses their negative emotions may be helped more by those around them (Graham et al., 2008).

Researchers study the effects of negative emotions on human behavior. Negative emotions indirectly influence prosocial and aggressive behaviors by modulating emotional self-efficacy, while a person’s depressive state exerts an inhibitory effect on self-efficacy to express positive emotions (Mesurado et al., 2018). One researcher investigated the relationship between adolescents’ sleep status and negative emotions. The results of the study showed that the shorter the sleep time, the lower the positive emotions and the stronger the negative emotions. Based on this, it is suggested that improving the quality of sleep can reduce emotion disorders (Shen et al., 2018). Negative emotions are a trigger for emotional eating in female college students. Some research suggests that experiential avoidance may help understanding the relationship between negative emotions and emotional eating in women. Experiential avoidance typically refers to the tendency to not want to maintain contact with aversive private experiences (Litwin et al., 2017). In addition, some studies have focused on the application of negative emotions in consumer behavior. It was found that negative emotions form part of the visitor experience. The experience of negative emotions can lead to a decrease in visitor satisfaction and also provide the possibility of transformation and self-development (Nawijn and Biran, 2018).

Few studies have examined the relationship between academic procrastination and negative emotions. However, some studies have focused on the relationship between procrastination itself and emotions of anxiety and fear. A study by Solomon and Rothblum (1984) suggested that fear of task failure and aversion to academic tasks are major factors contributing to students’ academic procrastination (Solomon and Rothblum, 1984). Therefore, schools should pay more attention to factors that can reduce student procrastination and their cognitive avoidance behaviors, which is likely to reduce students’ test anxiety (Firouzabad et al., 2018). People who procrastinate frequently may become sensitive to the anxiety it causes, which may trigger panic attacks (Hutchison et al., 2018). Several studies have also explored the relationship between academic procrastination and anxiety. One study used statistical methods to demonstrate that there was a direct and significant relationship between these two phenomena. The longer the students in the sample procrastinated, the higher their anxiety levels, which may also led to increased test anxiety (Araoz and Uchasara, 2020; Wang, 2021). Also, it has been noted that attention to and intervention in academic procrastination may help to reduce students’ test anxiety (Wang, 2021). There are also studies that focus on the aspect of negative emotions on academic procrastination. A study suggested that college students who have difficulty perceiving social support are more likely to have negative emotions in their daily lives. And they are more willing to engage in other irrelevant activities in order to have positive emotional experiences, which eventually leads to procrastination (Yang et al., 2021). And there are other researchers who believe that negative emotions motivate procrastination behaviors. Students reported more procrastination behaviors after experiencing high levels of negative emotions. However, procrastination behaviors did not predict changes in negative emotions (Pollack and Herres, 2020). Therefore, we propose the following hypothesis:

H1: There is a positive relationship between students’ academic procrastination and negative emotions.

### Online-Shopping Addiction

*Internet addiction* is an excessive or poorly controlled preoccupation, impulse, or behavior with Internet use that can lead to damage or suffering (Weinstein and Lejoyeux, 2010). Specific forms include the problematic use of the internet for such activities as the excessive viewing of internet videos or playing of online games (Montag et al., 2015). Scholars have developed different tools to assess such addictions. The Internet Addiction Inventory (CIAS) was developed by Chen et al. (2003). Other assessment tools have been created to identify and measure internet addiction in adolescents and adults (Huang et al., 2007; Aa et al., 2009; Meerkerk et al., 2009; Helmersson et al., 2011; Ang et al., 2012; Xu et al., 2012).

Internet addiction is directly related to online-shopping addiction (Seung-Hee and Won, 2005). Online-shopping addiction is defined as purchasing behavior that is out of one’s control due to lack of self-monitoring (LaRose and Eastin, 2002). One study found that online shopping was the strongest predictor of Internet addiction. In addition, social applications all significantly increase the odds of students being addicted to Internet use (Kuss et al., 2013). The concept that is relevant to the study of online shopping addiction is impulse buying. A study distinguishes between traditional impulsive buying behavior and online buying behavior and suggest that online buying behavior will become an alternative to traditional buying and should also receive attention from researchers (Akram et al., 2017).

A number of studies have now investigated the factors that influence online-shopping addiction. In one study, materialism was significantly and positively correlated with internet addiction, and a positive correlation was found between internet addiction and a tendency for impulsive online buying (Sun and Wu, 2011). There is also a relationship between online-shopping addictive behavior and hedonism (Günüç and Doğan Keskin, 2016; Doğan Keskin and Günüç, 2017). This hedonic impulsive behavior brings joy, relaxation, and happiness. Also, online platforms offering inexpensive products, a wide selection, and various promotions are related to addiction (Günüç and Doğan Keskin, 2016). Several researchers have developed tools for assessing online-shopping addictive behaviors, for example, the Online Shopping Addiction Scale (OSAS), which is based on the general addiction model (Duong and Liaw, 2021; Gong et al., 2021). The research of Zhao et al. (2017) shows that the scale has good reliability.

Few studies have focused on the relationship between academic procrastination and online-shopping addiction. However, some studies have focused on the relationship between general procrastination and Internet addiction or mobile phone use problems. Procrastination mitigates the effects of depression on internet addiction, and a high level of procrastination has a significant positive association with internet addiction (Hernández et al., 2019). At least one study has shown that procrastination is significantly and positively related to the problematic use of cell phones, and that students who procrastinated were more likely to use social media in class (Rozgonjuk et al., 2018). Procrastination may be the proximal cause of cell phone addiction in adolescents (Wang J. et al., 2019). In a study exploring the relationship between stress, internet addiction, and procrastination, procrastination had a significant positive relationship with internet addiction (Gong et al., 2021). From a different explanatory perspective, a study of student Facebook users (*N* = 345) suggested that procrastination is associated with low self-control. Both escapist values and procrastination lead individuals to choose temptations that produce pleasure; that is, both distracting one from the internet and also becoming more addicted to it (Meier et al., 2018). Trait procrastination was positively associated with adolescents performing online multitasking and having inadequate control over Internet use, and adolescents with high levels of trait procrastination may be at increased risk for procrastination due to inadequate control over Internet use (Reinecke et al., 2018). In addition, a survey of 483 college students indicated that cell phone addiction had a direct predictive effect on academic procrastination and an indirect predictive effect through academic self-efficacy. Specifically, academic self-efficacy partially mediated and buffered the effect between cell phone addiction and academic procrastination (Li et al., 2020).

In summary, it is reasonable to offer the following hypothesis:

H2: There is a positive relationship between academic procrastination and online-shopping addiction.

Currently, some studies have shown the relationship between online addiction and negative emotions, while some studies have also shown the relationship between shopping addiction and negative emotions. Users often feel guilty of procrastination when they overuse media (Panek, 2013). A study showed that smartphone addiction was positively related to adolescent depression (Wang P. et al., 2019). Hedonic online-shopping addiction can affect a person’s depression or even cause it to occur. Because of the monetary expenditures, interpersonal or family conflicts may occur (Doğan Keskin and Günüç, 2017). Internet addiction is seen as a mental health problem, with social anxiety avoidance behaviors being the strongest predictor of Internet addiction. A study has shown that internet addicts exhibit higher levels of social anxiety and depression, and lower self-esteem (Yucens and Uzer, 2018). Another study examined the effects of stress, social anxiety, and social class on adolescent Internet addiction and showed that Internet addiction was positively related to stress and social anxiety and negatively related to social class. Social class indirectly influenced Internet addiction by moderating the relationship between stress and social anxiety (Feng et al., 2019).

This review of the literature leads to the following hypothesis:

H3: There is a positive relationship between online-shopping addiction and negative emotions.

## Materials and Methods

### Research Design

To further investigate the mediating and buffering effects of online-shopping addiction on academic procrastination and negative emotions, freshman students in Guangdong Province were selected as participants in this study. Participants were in the preparation period for the college entrance exams, a highly stressful phase, during the pandemic outbreak. The questionnaire included four parts: demographic information, the Online Shopping Addiction Scale, the Academic Procrastination Scale, and the Depression-Anxiety-Stress Scale, around which the participants had developed their initial manifestations. Due to epidemic prevention and control restrictions, an online questionnaire was administered to participants. We used SPSS 25.0 software, Harman one-way test for common method bias to test the data, and Pearson correlation coefficient was calculated to test the variable relationships.

### Participants

This study was conducted in Guangdong Province, China. The inclusion criteria vertebrae of the participants were: adolescents who were preparing for college entrance examinations during the pandemic outbreak; and the exclusion criteria were: non-Guangdong freshman students, so as to ensure the homogeneity and representation of the sample drawn. Therefore, we chose stratified sampling in probability sampling. The sampling process was divided into three steps.

Step 1: 5 students distributed 450 questionnaires to first-year students in 10 vocational and technical colleges.

Step 2:430 students completed the questionnaire, and the researchers communicated with them to confirm that they were from Guangdong Province.

Step 3: The questionnaires of 423 students from Guangdong Province were retained for analysis.

The age range of this cohort of students ranged from 15 to 22 years old, with a mean age of 18.7 years old (SD: 0.86). Of these, 118 (27.9%) were males and 305 (72.1%) were females. Before the study design was finalized, the researchers had conducted exploratory focus-group interviews with five volunteers to clarify the possible association between academic procrastination and negative emotions in students. A majority of participants indicated that they had exhibited some level of anxiety or stress during the COVID-19 outbreak.

This study used a correlation design with an online questionnaire as the data collection method. The questionnaires were completed between November 10 and November 30, 2020. Five volunteers, either personally or by proxy, showed students the quick-response (QR) code for the questionnaire during breaks in an online university course. Students who volunteered scanned the QR code, went to the questionnaire screen, answered the questions, and then clicked on Submit. (A QR code is a readable barcode that contains information. A device such as a mobile phone or tablet scans the QR code with a camera, recognizes the binary data, and goes to a specific link. In China, QR codes are widely used to open specific link interfaces and various applications such as those for financial payments, identification, and information queries). It is important to emphasize that the purpose of the research were described in detail by the researcher before scanning the code, and all students filled out the questionnaire based on the voluntary principle.

### Material

The questionnaire used in this study consisted of four parts: demographic information, an Online-Shopping Addiction Scale, an Academic Procrastination Scale, and a Negative-Emotions Scale. The demographic information related to gender and age. The three scales were originally developed in English and were translated into Chinese for this study. To improve the quality of the translations, the back-translation method (Brislin, 1970) was used: the first researcher translated the English-language scale into Chinese, then the second researcher translated that version into English, after which the third researcher compared the original, translated, and back-translated versions to assess the accuracy of the translations. The translations were corrected and optimized before the questionnaire was finalized, which should have ensured the equivalence of the scales.

#### Online Shopping Addiction Scale

This study used the Online Shopping Addiction Scale developed by Zhao et al. (2017). The scale includes 17 self-report items. Each item is rated on a 5-point Likert scale (1 = totally disagree, 2 = disagree, 3 = neither agree nor disagree, 4 = agree, 5 = totally agree). Possible scores range from 18 to 90. In this study, the internal consistency coefficient for the scale was 0.951.

#### Tuckman Academic Procrastination Scale

This study used the Tuckman Academic Procrastination Scale developed by Tuckman (1991). This scale consists of 16 questions that measure a person’s level of academic procrastination. In order to maintain consistency, this scale was converted from a 4-point to a 5-point scale, ranging from 1 (completely disagree) to 5 (completely agree). The conversion resulted in a range of possible scores of from 16 to 80. In the current study, the internal consistency coefficient of the scale was 0.917.

#### Depression-Anxiety-Stress Scale

This study used the Depression-Anxiety-Stress Scale, as revised by Antony et al. (1998), to measure negative emotions. The scale has 21 items divided among three dimensions: depression, anxiety, and stress. To maintain consistency, this scale was shifted from a 4-point to a 5-point scale, ranging from 1 (completely disagree) to 5 (completely agree), so that possible scores ranged from 21 to 105. In the current study, the internal consistency coefficient of the scale was 0.959.

### Data Analysis

SPSS 25.0 was used to process and analyze the data. The data collected were self-reported, and so to ensure the validity, the common method biases were tested before data processing using Harman’s one-way test (Podsakoff et al., 2003). The testing examined the 54 items in the questionnaire related to three variables. The results showed that 8 factors had eigenvalues greater than 1. The contribution of the 8 factors to the total variance was 70.14%, and the variance explained by the first factor was only 30.113%, which did not reach the critical criterion of 40% (Zhou and Long, 2004). Thus, there was no significant common method bias in this study.

We next performed descriptive analysis, correlation analysis, and model testing of the data based on the research hypotheses. First, we examined the concentration and dispersion trends of the data through descriptive analysis. Then, we conducted correlation analysis among the variables to test the relationships among the independent, mediating, dependent, and moderating variables by calculating Pearson correlation coefficients. Based on the correlations, the research hypotheses were further tested by using the PROCESS (version 3.3) plug-in in SPSS to test the model’s mediating and moderating effects. (The PROCESS plug-in was developed by Hayes (2013) specifically for path analysis-based moderating and mediating analyses and their combinations).

## Results

### Descriptive and Correlation Analysis

The results of the descriptive analysis of academic procrastination, online-shopping addiction and negative emotions are shown in Table 1. The mean for academic procrastination was 45.5 with a standard deviation of 10.8; the mean for online-shopping addiction was 37.9 with a standard deviation of 12.0; and the mean for negative emotions was 48.5 with a standard deviation of 16.0. When the sample scores were above the mean, high levels of procrastination and addictive behavior were observed. When the sample scores were below the mean, there was a low level of procrastination and addictive behavior. Thus, the sample had varying degrees of procrastination and addictive behavior.

**TABLE 1:** Descriptive statistics and Pearson’s r for the variables.

Variables	*N*	*M*	*SD*	Procrastination	Online-shopping addiction	Negative emotions
Procrastination	423	45.5	10.8	1		
Online-shopping addiction	423	37.9	12.0	0.176**	1	
Negative emotions	423	48.5	16.0	0.250**	0.358**	1

**p < 0.01.

The correlations of the three variables were assessed using Pearson’s product difference correlation coefficient, and the results are presented in Table 1. First, academic procrastination was significantly and positively correlated with online-shopping addiction (*r* = 0.176, *p* < 0.01). Second, academic procrastination was significantly and positively correlated with negative emotions (*r* = 0.250, *p* < 0.01). Third, online-shopping addiction was significantly and positively associated with negative emotions (*r* = 0.358, *p* < 0.01).

### Analysis of Mediating Effects

The PROCESS (version 3.3) software (model 4) was used to test the mediating effect of online-shopping addiction between the two variables, using academic procrastination as the independent variable and negative emotions as the dependent variable. The results indicated that academic procrastination significantly predicted negative emotions (β = 0.3716, *t* = 5.2888, *p* < 0.001). The predictive effect remained significant after adding online-shopping addiction as a mediating variable (β = 0.2819, *t* = 4.1744, *p* < 0.001). Academic procrastination had a significant positive predictive effect on online-shopping addiction (β = 0.1955, *t* = 3.8490, *p* < 0.001). The positive predictive effect remained significant after adding gender as a mediating variable (β = 4.1795, *t* = 3.2985, *p* < 0.001). Online-shopping addiction had a significant positive predictive effect on negative emotions (β = 0.4416, *t* = 7.2006, *p* < 0.001) (see Table 2). In addition, both the direct effect of academic procrastination on negative emotions and the mediating effect of online-shopping addiction had bootstrap confidence intervals (95%) with no zero between the lower and upper bounds (see Table 3).

**TABLE 2:** Mediation analysis results for the three variables.

Regression equation		Fitting indices				Significance
Outcome variables	Predictor variables	*R*	*R* ^2^	*F(df)*	β	*T*
Online-shopping addiction	Procrastination	0.2353	0.0553	12.3031***	0.1955	3.8490***
	Gender (M-F)				4.1795	3.2985***
Negative emotions	Online-shopping addiction	0. 4070	0.1657	27.7360***	0. 4416	7.2006***
	Procrastination				0. 2819	4.1744***
	Gender (M-F)				–1.5093	–0.9358
Negative emotions	Procrastination	0.2499	0.0624	13.9863***	0. 3716	5.2888***
	Gender (M-F)				0.3362	0.1994

***p < 0.001.

**TABLE 3:** Total effect, direct effect, and indirect effect among the variables.

	Effect size	Boot SE	Boot CI lower limit	Boot CI upper limit	Relative effect size
Total effect	0.3710	0.0701	0.2332	0.5088	100.00%
Direct effect	0.2865	0.0673	0.1541	0.4188	77.22%
Indirect effect	0.0845	0.0306	0.0324	0.1542	22.78%

*Boot CI, Boot confidence interval. Lower limit and upper limit = The lower limit and upper limit of 95% Boot confidence interval. Relative effect size = Direct effect size or indirect effect size divided by total effect size.*

## Discussion

### Discussion of the Results

In this study, we developed a moderating mediator model of the relationship between academic procrastination and negative emotions in adolescents during the COVID-19 pandemic. It was found that (a) a positive relationship existed between students’ academic procrastination and negative emotions; (b) a positive relationship existed between academic procrastination and online-shopping addiction; (c) a positive relationship existed between online-shopping addiction and negative emotions; and (d) the online-shopping addiction mediated and buffered the relationship between academic procrastination and negative emotions. These results are consistent with the proposed hypothesis and previous findings.

First, the findings are consistent with H1 and the existing literature, revealing that academic procrastination is positively associated with negative emotions. Sirois and Pychyl (2013) argued that procrastination may be best understood as a form of self-regulation failure that involves the primacy of short-term emotion repair and emotion regulation over the longer-term pursuit of intended actions. Rahimi and Vallerand (2021) demonstrated that, during the COVID-19 pandemic, negative emotions can be positively associated with academic procrastination, while positive emotions can prevent it, so people choose to focus on immediate happiness during a pandemic (Rahimi and Vallerand, 2021). On the other hand, strong and consistent predictors of procrastination were task aversiveness, and its facets of self-control, distractibility, organization, and achievement motivation (Steel, 2007). If the person considers that they do not have the skills required to carry out a task and be successful, they will be more likely to postpone that task in order to prevent this skill deficit from manifesting (Quant and Sánchez, 2012). Further, symptoms of panic disorder, social anxiety disorder, and health anxiety have been associated with the level of procrastination (Hutchison et al., 2018), and Araoz and Uchasara’s (2020) showed that the longer the period of procrastination, the higher the level of anxiety.

The results of the present study also validated H2. First, the findings showed a positive relationship between academic procrastination and online-shopping addiction, which is consistent with the results of previous studies (Rozgonjuk et al., 2018). Yang et al. (2019) suggested that destructive academic emotions and behaviors among university students, such as anxiety and procrastination, might partly be a consequence of poor self-control concerning their smartphone use. Procrastination may well be the result of an unwillingness to stop pleasure-seeking activities using smartphones online or offline. Individuals suffering from smartphone addiction may find it particularly difficult to stop using this device or to manage its disruptions (Zhang and Wu, 2020). This leads to procrastination before adolescents turn to their studies. Second, procrastination as a personality trait may be a risk factor for mobile-phone addiction. Procrastinators are characterized by low self-control and a preference for short-term rewards, and they are prone to become internet addicts because of the design features of their devices (Yang et al., 2021).

More specifically, self-regulation can be used as an explanatory mechanism for this effect. Procrastinators are unable to control their behavior and prefer to surrender important goals in favor of pleasurable short-term activities (Ferrari et al., 1995). Sirois and Pychyl argues that procrastination is strongly associated with short-term emotional repair and emotion regulation (Sirois and Pychyl, 2013). Aversive tasks can lead to anxiety, and avoiding them is a way to escape this negative emotion (Tice and Bratslavsky, 2000). The negative emotions that people experience after failure can also influence self-regulatory beliefs and behaviors, and performance goals. This would explain the different ability of different students to tolerate mistakes in the learning process (Turner et al., 1998). In conclusion, in order to prevent and reduce these behaviors the school should focus on learning and test anxiety, motivation and emotion regulation issues in adolescents.

The results are also consistent with H3. Studies have shown that hedonic shopping addiction can lead to a negative emotions or even to depression. Because of online-shopping addiction and its associated financial costs, interpersonal or family conflicts may occur (Doğan Keskin and Günüç, 2017). Impulse buying is fundamentally a problem of failing to delay gratification. This leads to worse emotions, as it is still yourself who bears the consequences in the future (Sirois and Pychyl, 2013). Through the shopping excitement index prediction experiment, the buying behavior is described as used to regulate emotions, alleviate or escape negative emotions. Therefore, we think online-shopping can be addictive (Trotzke et al., 2015). The desire to shop is replaced by feelings of guilt, and the pleasure gained from shopping quickly disappears: compulsive shoppers seem to cycle between full attention, ritualization, compulsive buying, and despair (Clark and Calleja, 2009).

Finally, From the above we can see the findings suggest that online-shopping addiction mediates and buffers academic procrastination and negative emotions. A study based on the compensatory-internet-use model and emotion-regulation theory concluded that procrastination mediates the relationship between perceived stress and internet addiction (Gong et al., 2021), which suggests that this article echoes the findings of previous studies. These results also provide a perspective on the possible cause of internet addiction among college students; namely, that individuals use the internet to avoid stress and to procrastinate (Hernández et al., 2019). That is, individuals who have procrastinated are more likely to have a cell phone addiction caused by stress (Wang J. et al., 2019), because people in negative emotions tend to engage in greater subsequent self-gratification and self-reward than people in neutral emotions (Tice and Bratslavsky, 2000). As the priority theory of short-term emotion regulation says that emotion regulation the processes underlying procrastination are driven by a need to regulate the emotion of the present self at the expense of the future self (Sirois and Pychyl, 2013). So online-shopping addiction can regulate and buffer against academic delays and negative emotions.

### Implications

Theoretically, we have established a link between academic procrastination and negative emotions and deepened understanding of the impact of academic procrastination on negative emotions. In addition, we have shown that online-shopping addiction can mediate and buffer the effects of academic procrastination on negative emotions. This finding suggests that college students who are academic procrastinators can slow down negative emotions by controlling their online-shopping behavior. This could enhance their ability to cope with challenges. Practically, the relationship between the three variables we proposed can help researchers better understand the mechanisms of academic procrastination and its impact on negative emotions among college students, thus providing support for the improvement of college students’ learning effectiveness.

### Limitations and Future Directions

There are some limitations in this study. First, this study used a cross-sectional design and the study participants were all from one university in Guangdong Province, so the study sample limits the universality of the findings. Future researchers could use a longitudinal research design and try to recruit participants from different regions and institutions. Second, the scale measures students’ depression, anxiety, and stress. The present study considered negative emotional problems with the combination of these three. Future research could focus on the relationship between depression, anxiety or stress and the remaining two variables, which may lead to new findings. In addition, the range of negative emotions measured in this study was broad, and appropriate instruments could be selected to measure academic emotions in the future. Indirect and mediating effects are weakly represented in the study. Therefore optimizing the conceptual model structure is also a direction we will continue to investigate in depth in the future. What’s more, we could explore other possible mediating variables that have an impact on academic procrastination and negative emotions. Research is also needed to investigate how to guide students to minimize academic-procrastination behaviors in order to alleviate or reduce negative emotions.

## Conclusion

We explored the relationship between the academic procrastination, negative emotions, and online-shopping addiction of college students during COVID-19. The results indicate that the level of academic procrastination positively influenced their levels of online-shopping addiction and negative emotions, and their level of online-shopping addiction positively influenced their level of negative emotions. In addition, there was a mediating effect between the degree of online-shopping addiction and the degree of academic procrastination and negative emotions. In other words, with the mediating effect of online-shopping addiction, the higher the level of students’ academic procrastination, the more likely they were to have a high score for negative emotions.

## Data Availability Statement

The original contributions presented in the study are included in the article/supplementary material, further inquiries can be directed to the corresponding author/s.

## Ethics Statement

The studies involving human participants were reviewed and approved by the Ethics Committee of Capital Normal University. The patients/participants provided their written informed consent to participate in this study.

## Author Contributions

KW collected the data. QW and YX designed the research and analyzed the data. QW and ZK reviewed the literature and edited the manuscript. QW, YX, ZK, YD, and KW wrote the manuscript. All authors have read and agreed to the published version of the manuscript.

## Conflict of Interest

The authors declare that the research was conducted in the absence of any commercial or financial relationships that could be construed as a potential conflict of interest.

## Publisher’s Note

All claims expressed in this article are solely those of the authors and do not necessarily represent those of their affiliated organizations, or those of the publisher, the editors and the reviewers. Any product that may be evaluated in this article, or claim that may be made by its manufacturer, is not guaranteed or endorsed by the publisher.

## References

[B1] Aa N. Overbeek G. Engels R. Scholte R. Meerkerk G. J. Eijnden R. J. J. M. V. D. (2009). Daily and Compulsive Internet Use and Well-Being in Adolescence: A Diathesis-Stress Model Based on Big Five Personality Traits. *J. Youth Adolesc.* 38:765. 10.1007/s10964-008-9298-3 19636779

[B2] Akram U. Hui P. Khan M. K. Hashim M. Saduzai S. K. (2017). *Impulsive Buying: A Qualitative Investigation of the Phenomenon.* Singapore: Springer, 1383–1399.

[B3] Alyami H. S. Naser A. Y. Dahmash E. Z. Alyami M. H. Alyami M. S. (2021). Depression and anxiety during the COVID-19 pandemic in Saudi Arabia: a cross-sectional study. *Int. J. Clin. Pract.* 75:e14244. 10.1111/ijcp.14244 33876864 PMC8249996

[B4] Ang R. P. Chong W. H. Chye S. Huan V. S. (2012). Loneliness and generalized problematic Internet use: parents’ perceived knowledge of adolescents’ online activities as a moderator. *Comput. Hum. Behav.* 28 1342–1347. 10.1016/j.chb.2012.02.019

[B5] Antony M. M. Bieling P. J. Cox B. J. Enns M. W. Swinson R. P. (1998). Psychometric properties of the 42-item and 21-item versions of the Depression Anxiety Stress Scales in clinical groups and a community sample. *Psychol. Assessm.* 10 176–181. 10.1037/1040-3590.10.2.176

[B6] Araoz E. Uchasara H. (2020). Academic procrastination and anxiety among university students in Madre de Dios, Peru. *Apuntes Univers.* 10 322–337.

[B7] Brislin R. W. (1970). Back-Translation for Cross-Cultural Research. *J. Cross Cult. Psychol.* 1 185–216. 10.1177/135910457000100301

[B8] Chen S. H. Weng L. J. Su Y. J. Wu H. M. Yang P. F. (2003). Development of a Chinese internet addiction scale and its psychometric study. *Chin. J. Psychol.* 45 279–294.

[B9] Chun Chu A. H. Choi J. N. (2005). Rethinking procrastination: positive effects of “active” procrastination behavior on attitudes and performance. *J. Soc. Psychol.* 145 245–264. 10.3200/SOCP.145.3.245-264 15959999

[B10] Clark M. Calleja K. (2009). Shopping addiction: a preliminary investigation among Maltese university students. *Addict. Res. Theory* 16 633–649. 10.1080/16066350801890050

[B11] Closson L. M. Boutilier R. R. (2017). Perfectionism, academic engagement, and procrastination among undergraduates: The moderating role of honors student status. *Learn. Individ. Differ.* 57 157–162. 10.1016/j.lindif.2017.04.010

[B12] Diener E. Larsen R. J. Levine S. Emmons R. A. (1985). Intensity and frequency: dimensions underlying positive and negative affect. *J. Pers. Soc. Psychol.* 48 1253–1265. 10.1037/0022-3514.48.5.1253 3998989

[B13] Dietz F. Hofer M. Fries S. (2011). Individual values, learning routines and academic procrastination. *Br. J. Educ. Psychol.* 77 893–906. 10.1348/000709906X169076 17971288

[B14] Doğan Keskin A. Günüç S. (2017). Testing models regarding online shopping addiction. *Addicta* 4 221–242. 10.15805/addicta.2017.4.2.0010

[B15] Duong X. L. Liaw S. Y. (2021). Psychometric evaluation of Online Shopping Addiction Scale (OSAS). *J. Hum. Behav. Soc. Environ.* 11 1–11. 10.1080/10911359.2021.1938330

[B16] Feng Y. Ma Y. Zhong Q. (2019). The Relationship Between Adolescents’ Stress and Internet Addiction: A Mediated-Moderation Model. *Front. Psychol.* 10:2248. 10.3389/fpsyg.2019.02248 31636590 PMC6787273

[B17] Ferrari J. R. Johnson J. L. McCown W. G. (1995). *Procrastination and task avoidance : Theory, research, and treatment.* New York, NY: Plenum.

[B18] Firouzabad Y. H. Nejad S. B. Davoudi I. (2018). Prediction of Subscale Test Anxiety Considering Behavioral Procrastination, Decisional Procrastination and Cognitive Avoidance in University Students. *Iran. J. Psychiatry Clin. Psychol.* 23 424–437. 10.29252/nirp.ijpcp.23.4.424

[B19] Fredrickson B. (2001). The role of positive emotions in positive psychology: the broaden-and-build theory of positive emotions. *Am. Psychol.* 56 218–226. 10.1037/0003-066X.56.3.218 11315248 PMC3122271

[B20] Gareau A. Chamandy M. Kljajic K. Gaudreau P. (2019). The detrimental effect of academic procrastination on subsequent grades: the mediating role of coping over and above past achievement and working memory capacity. *Anxiety Stress Coping* 32 141–154. 10.1080/10615806.2018.1543763 30406682

[B21] Gong Z. Wang L. Wang H. (2021). Perceived stress and internet addiction among Chinese college students: mediating effect of procrastination and moderating effect of flow. *Front. Psychol*. 12:632461. 10.3389/fpsyg.2021.632461 34262501 PMC8273309

[B22] Goroshit M. Hen M. (2019). Academic procrastination and academic performance: Do learning disabilities matter? *Curr. Psychol.* 40 2490–2498. 10.1007/s12144-019-00183-3

[B23] Graham S. M. Huang J. Y. Clark M. S. Helgeson V. S. (2008). The positives of negative emotions: willingness to express negative emotions promotes relationships. *Pers. Soc. Psychol. Bull*. 34 394–406. 10.1177/0146167207311281 18272807

[B24] Günüç S. Doğan Keskin A. (2016). Online shopping addiction: symptoms, causes and effects. *Addicta* 3 353–364. 10.15805/addicta.2016.3.0104

[B25] Hayes A. F. (2013). *Introduction to Mediation, Moderation, and Conditional Process Analysis: A Regression-Based Approach.* New York, NY: Guilford Press.

[B26] Helmersson B. K. Anders B. Olle F. (2011). Extensive Internet Involvement—Addiction or Emerging Lifestyle? *Int. J. Environ. Res. Public Health* 8:ijerh8124488. 10.3390/ijerph8124488 22408585 PMC3290978

[B27] Hernández C. Rivera Ottenberger D. Moessner M. Crosby R. D. Ditzen B. (2019). Depressed and swiping my problems for later: The moderation effect between procrastination and depressive symptomatology on internet addiction. *Comput. Human Behav.* 97:1–9. 10.1016/j.chb.2019.02.027

[B28] Hofmann S. G. (2021). The impact of COVID-19 on mental health. *Cogn. Behav. Ther.* 50 185–190. 10.1080/16506073.2021.1897666 34018470

[B29] Huang Z. Wang M. O. Qian M. Zhong J. Tao R. (2007). Chinese Internet Addiction Inventory: developing a measure of problematic internet use for Chinese college students. *Cyberpsychol. Behav.* 10 805–812. 10.1089/cpb.2007.9950 18085968

[B30] Hutchison T. Penney A. M. Crompton J. (2018). Procrastination and anxiety: exploring the contributions of multiple anxiety-related disorders. *Curr. Iss. Personal. Psychol.* 6 122–129. 10.5114/CIPP.2018.73054

[B31] Jia J. Wang L.-I. Xu J.-B. Lin X.-H. Zhang B. Jiang Q. (2021). Self-Handicapping in Chinese Medical Students During the COVID-19 Pandemic: The Role of Academic Anxiety, Procrastination and Hardiness. *Front. Psychol.* 12:741821. 10.3389/fpsyg.2021.741821 34603160 PMC8484870

[B32] Kandemir M. (2014). Reasons of academic procrastination: self-regulation, academic self-efficacy, life satisfaction and demographics variables. *Proc. Soc. Behav. Sci.* 2014 188–193. 10.1016/j.sbspro.2014.09.179

[B33] Kuss D. J. Griffiths M. D. Binder J. F. (2013). Internet addiction in students: Prevalence and risk factors. *Comput. Hum. Behav.* 29 959–966. 10.1016/j.chb.2012.12.024

[B34] LaRose R. Eastin M. S. (2002). Is online buying out of control? Electronic commerce and consumer self-regulation. *J. Broadcast. Electron. Media* 46 549–564. 10.1207/s15506878jobem4604_4

[B35] Li L. Gao H. Xu Y. (2020). The mediating and buffering effect of academic self-efficacy on the relationship between smartphone addiction and academic procrastination. *Comput. Educat.* 159:104001. 10.1016/j.compedu.2020.104001

[B36] Litwin R. Goldbacher E. M. Cardaciotto L. Gambrel L. E. (2017). Negative emotions and emotional eating: the mediating role of experiential avoidance. *Eat Weight Disord.* 22 97–104. 10.1007/s40519-016-0301-9 27460010

[B37] Liu W. Pan Y. Luo X. Wang L. Pang W. (2017). Active procrastination and creative ideation: The mediating role of creative self-efficacy. *Personal. Individ. Differ.* 119 227–229. 10.1016/j.paid.2017.07.033

[B38] Meerkerk G. J. Van Den Eijnden R. J. J. M. Vermulst A. A. Garretsen H. (2009). The Compulsive Internet Use Scale (CIUS): some psychometric properties. *CyberPsychol. Behav.* 12 1–6. 10.1089/cpb.2008.0181 19072079

[B39] Meier A. Meltzer C. Reinecke L. (2018). “Coping with stress or losing control? Facebook-induced strains among emerging adults as a consequence of escapism versus procrastination,” in *Youth and media: current perspectives on media use and effects*, eds Kühne R. Baumgartner S. E. Koch T. Hofer M. (Baden-Baden: Nomos), 167–186. 10.5771/9783845280455-167

[B40] Mesurado B. Vidal E. M. Mestre A. L. (2018). Negative emotions and behaviour: The role of regulatory emotional self-efficacy. *J. Adolesc.* 64 62–71. 10.1016/j.adolescence.2018.01.007 29408100

[B41] Montag C. Bey K. Sha P. Li M. Chen Y. F. Liu W. Y. (2015). Is it meaningful to distinguish between generalized and specific Internet addiction? evidence from a cross-cultural study from Germany, Sweden, Taiwan and China. *Asia Pac. Psychiatry* 7 20–26. 10.1111/appy.12122 24616402

[B42] Nawijn J. Biran A. (2018). Negative emotions in tourism: a meaningful analysis. *Curr. Iss. Tourism* 22 2386–2398. 10.1080/13683500.2018.1451495

[B43] Nordby K. Klingsieck K. B. Svartdal F. (2017). Do procrastination-friendly environments make students delay unnecessarily? *Soc. Psychol. Educat.* 20 491–512. 10.1007/s11218-017-9386-x

[B44] Panek E. (2013). Left to Their Own Devices. *Commun. Res.* 41 561–577. 10.1177/0093650213499657

[B45] Peixoto E. M. Pallini A. C. Vallerand R. J. Rahimi S. Silva M. V. (2021). The role of passion for studies on academic procrastination and mental health during the COVID-19 pandemic. *Soc. Psychol. Educ*. 8 1–17. 10.1007/s11218-021-09636-9 34121913 PMC8184402

[B46] Podsakoff P. M. MacKenzie S. B. Lee J. Y. Podsakoff N. P. (2003). Common method biases in behavioral research: A critical review of the literature and recommended remedies. *J. Appl. Psychol.* 88 879–903. 10.1037/0021-9010.88.5.879 14516251

[B47] Pollack S. Herres J. (2020). Prior Day Negative Affect Influences Current Day Procrastination: A Lagged Daily Diary Analysis. *Anxiety Stress Coping* 33 165–175. 10.1080/10615806.2020.1722573 32008351

[B48] Qiu J. Shen B. Zhao M. Wang Z. Xie B. Xu Y. (2020). A nationwide survey of psychological distress among Chinese people in the COVID-19 epidemic: implications and policy recommendations. *Gen. Psychiatr.* 33:e100213. 10.1136/gpsych-2020-100213 32215365 PMC7061893

[B49] Quant D. M. Sánchez A. (2012). Procrastinación, procrastinación académica: concepto e implicaciones. *Rev. V Anguardia Psicol. Clín. Teórica Práct.* 3 45–59.

[B50] Rahimi S. Vallerand R. (2021). The role of passion and emotions in academic procrastination during a pandemic (COVID-19). *Pers. Individ.Dif.* 179:110852. 10.1016/j.paid.2021.110852

[B51] Ramzi F. Saed O. (2019). The Roles of Self-Regulation and Self-Control in Procrastination. *Psychol. Behav. Sci. Int. J.* 13:555863. 10.19080/PBSIJ.2019.13.555863

[B52] Reinecke L. Meier A. Beutel M. E. Schemer C. Stark B. Wolfling K. (2018). The Relationship Between Trait Procrastination, Internet Use, and Psychological Functioning: Results From a Community Sample of German Adolescents. *Front. Psychol.* 9:913. 10.3389/fpsyg.2018.00913 29942268 PMC6004405

[B53] Rowe A. D. Fitness J. (2018). Understanding the Role of Negative Emotions in Adult Learning and Achievement: A Social Functional Perspective. *Behav. Sci.* 8:bs8020027. 10.3390/bs8020027 29461487 PMC5836010

[B54] Rozgonjuk D. Kattago M. Täht K. (2018). Social media use in lectures mediates the relationship between procrastination and problematic smartphone use. *Comput. Human Behav.* 89 191–198. 10.1016/j.chb.2018.08.003

[B55] Seung-Hee L. Won J. J. (2005). Research Model for Internet Shopping Addictive Buying on Fashion Products: Mediating Effect of Internet Addiction. *J. Kor. Soc. Clothing Textiles* 29 167–176.

[B56] Shen L. van Schie J. Ditchburn G. Brook L. Bei B. (2018). Positive and Negative Emotions: Differential Associations with Sleep Duration and Quality in Adolescents. *J. Youth Adolesc.* 47 2584–2595. 10.1007/s10964-018-0899-1 30039509

[B57] Shih S.-S. (2016). Factors related to Taiwanese adolescents’ academic procrastination, time management, and perfectionism. *J. Educat. Res.* 110 415–424. 10.1080/00220671.2015.1108278

[B58] Sirois F. Pychyl T. (2013). Procrastination and the priority of short-term mood regulation: consequences for future self. *Soc. Pers. Psychol. Compass* 7 115–127. 10.1111/spc3.12011

[B59] Solomon L. J. Rothblum E. D. (1984). Academic procrastination: frequency and cognitive-behavioral correlates. *J. Couns. Psychol.* 31 503–510. 10.1037/0022-0167.31.4.503

[B60] Stead R. Shanahan M. J. Neufeld R. (2010). ”I’ll go to therapy, eventually”: procrastination, stress and mental health. *Pers. Indiv. Dif.* 49 175–180. 10.1016/j.paid.2010.03.028

[B61] Steel P. (2007). The nature of procrastination: a meta-analytic and theoretical review of quintessential self-regulatory failure. *Psychol. Bull.* 133 65–94. 10.1037/0033-2909.133.1.65 17201571

[B62] Steel P. (2010). Arousal, avoidant and decisional procrastinators: do they exist? *Pers. Indiv. Dif.* 48 926–934. 10.1016/j.paid.2010.02.025

[B63] Sun T. Wu G. (2011). Trait predictors of online impulsive buying tendency: a hierarchical approach. *J. Mark. Theory Pract.* 19 337–346. 10.2753/MTP1069-6679190307

[B64] Tice D. M. Bratslavsky E. (2000). Giving in to Feel Good: The Place of Emotion Regulation in the Context of General Self-Control. *Psychol. Inquiry* 11 149–159. 10.1207/S15327965PLI1103_03 26627889

[B65] Trotzke P. Starcke K. Müller A. Brand M. (2015). Pathological buying online as a specific form of internet addiction: a model-based experimental investigation. *PLoS One* 10:0140296. 10.1371/journal.pone.0140296 26465593 PMC4605699

[B66] Tuckman B. W. (1991). The Development and Concurrent Validity of the Procrastination Scale. *Educat. Psychol. Measure.* 51 473–480.

[B67] Turner J. C. Thorpe P. K. Meyer D. K. (1998). Students’ reports of motivation and negative affect: A theoretical and empirical analysis. *J. Educ. Psychol.* 90 758–771. 10.1037/0022-0663.90.4.758

[B68] Wang J. Wang P. Yang X. Zhang G. Wang X. Zhao F. (2019). Fear of missing out and procrastination as mediators between sensation seeking and adolescent smartphone addiction. *Int. J. Ment. Health Addict.* 17 1049–1062. 10.1007/s11469-019-00106-0

[B69] Wang P. Liu S. Zhao M. Yang X. Zhang G. Chu X. (2019). How is problematic smartphone use related to adolescent depression? A moderated mediation analysis. *Children Youth Serv. Rev.* 104:104384. 10.1016/j.childyouth.2019.104384

[B70] Wang Y. (2021). Academic procrastination and test anxiety: a cross-lagged panel analysis. *J. Psychol. Couns. Sch.* 31 122–129. 10.1017/jgc.2020.29

[B71] Watson D. Clark L. A. Tellegen A. (1988). Development and validation of brief measures of positive and negative affect: the PANAS scales. *J. Pers. Soc. Psychol.* 54 1063–1070. 10.1037/0022-3514.54.6.1063 3397865

[B72] Weinstein A. Lejoyeux M. (2010). Internet Addiction or Excessive Internet Use. *Am. J. Drug Alcohol Abuse* 36 277–283. 10.3109/00952990.2010.491880 20545603

[B73] Wolters C. A. (2003). Understanding procrastination from a self-regulated learning perspective. *J. Educ. Psychol.* 95 179–187. 10.1037/0022-0663.95.1.179

[B74] Xu J. Shen L. X. Yan C. H. Hu H. Yang F. Wang L. (2012). Personal characteristics related to the risk of adolescent internet addiction: a survey in Shanghai, China. *BMC Public Health* 12:1106. 10.1186/1471-2458-12-1106 23259906 PMC3563549

[B75] Yang X. F. Zhu J. R. Hu P. (2021). Perceived social support and procrastination in college students: A sequential mediation model of self-compassion and negative emotions. *Curr. Psychol.* 9:3. 10.1007/s12144-021-01920-3

[B76] Yang Z. Asbury K. Griffiths M. (2019). An Exploration of Problematic Smartphone Use among Chinese University Students: Associations with Academic Anxiety, Academic Procrastination, Self-Regulation and Subjective Wellbeing. *Int. J. Mental Health Addict.* 17 596–614. 10.1007/s11469-018-9961-1

[B77] Yucens B. Uzer A. (2018). The relationship between internet addiction, social anxiety, impulsivity, self-esteem, and depression in a sample of Turkish undergraduate medical students. *Psychiatry Res.* 267 313–318. 10.1016/j.psychres.2018.06.033 29957547

[B78] Zhang M. X. Wu A. (2020). Effects of smartphone addiction on sleep quality among Chinese university students: The mediating role of self-regulation and bedtime procrastination. *Addict. Behav.* 111:106552. 10.1016/j.addbeh.2020.106552 32717501

[B79] Zhang Y. Ma Z. F. (2020). Impact of the COVID-19 pandemic on mental health and quality of life among local residents in Liaoning Province, China: A cross-sectional study. *Int. J. Environ. Res. Public Health* 17:2381. 10.3390/ijerph17072381 32244498 PMC7177660

[B80] Zhao H. Tian W. Tao X. (2017). The Development and Validation of the Online Shopping Addiction Scale. *Front. Psychol.* 8:735. 10.3389/fpsyg.2017.00735 28559864 PMC5432625

[B81] Zhou H. Long L. (2004). Statistical test and control method for common method deviation. *Adv. Psychol. Sci.* 12 942–942.

